# Eosinophilic Panniculitis and Insect Bite-Like Eruption in a Patient with Chronic Lymphocytic Leukaemia: A Spectrum of the Same Entity

**DOI:** 10.1155/2010/263827

**Published:** 2010-06-07

**Authors:** R. Rodríguez-Lojo, M. Almagro, F. Piñeyro, L. Pérez-Varela, B. Fernández-Jorge, J. Del Pozo, F. Sacristán, E. Fonseca

**Affiliations:** ^1^Department of Dermatology, Complexo Hospitalario Universitario A Coruña, 15006 A Coruña, Spain; ^2^Department of Pathology, Complexo Hospitalario Universitario A Coruña, 15006 A Coruña, Spain

## Abstract

*Background*. Eosinophilic dermatosis of hematologic disorders is a reactive process that may cause a variety of clinical manifestations. *Methods*. We report a patient who had outbreaks of skin lesions since the onset of chronic lymphocytic leukaemia. *Results*. The cutaneous eruptions began as eosinophilic panniculitis and after changed to insect bite-like lesions. *Conclusion*. We think that eosinophilic panniculitis and insect bite-like lesions may be part of the spectrum of the same entity in patients with hematologic disorders.

## 1. Introduction

Eosinophilic panniculitis is an unusual type of panniculitis characterized by a prominent infiltration of subcutaneous fat with eosinophils. It is a reactive pattern that may be associated with a variety of conditions such as arthropod bites, parasitic infestations, drugs reactions, vasculitis, neoplasm, or hematologic diseases [1, 2].

We report a case of eosinophilic panniculitis in a patient with chronic lymphocytic leukaemia (CLL), in which the morphology of cutaneous eruption changed later to insect bite-like lesions.

## 2. Case Report

**Figure 1:**
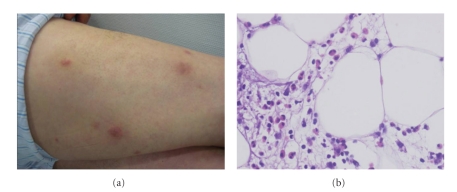
(a) Inflammatory nodules on thighs. (b) Panniculitis with eosinophils infiltration.

A 63-year-old man was diagnosed of chronic lymphoproliferative syndrome in 2006, classified as chronic lymphocytic leukaemia (CLL) of B cells and treated with chlorambucil for 9 months; fludarabine for 4 months; cyclophosphamide, vincristine, prednisone, and rituximab (CVP-R) for 3 months; cyclophosphamide, doxorubicin, vincristine, and prednisone (CHOP) for 6 months. In November 2007, he consulted for a 1-year history of recurrent, painful nodules with inflammatory signs on his limbs and trunk (Figure 1(a)). This skin eruption had appeared since onset of the CLL and cutaneous lesions sometimes worsened after chemotherapy cycles. Individual lesions showed a typical course of one-week duration and spontaneous resolution without scar formation. There were not constitutional symptoms or mucosal lesions.

The histopathologic study of a cutaneous specimen showed an inflammatory infiltrate predominantly composed by eosinophils that involved both the fat lobules and the septa (Figure 1(b)). No changes in dermis or epidermis were observed. The results of laboratory studies were normal except for an eosinophilic count of 0.69 × 10^9^/L (8.6%). The remaining laboratory findings (elevated *β*_2_ microglobulin level and polymorphous lymphocytes in peripheral blood smears) were consistent with his haematological condition. Other possible causes of eosinophilic tissue infiltration were ruled out, and he was diagnosed of eosinophilic panniculitis related to his hematologic disease. Skin nodules disappeared after a short course of systemic steroids, and chemotherapy was changed to CHOP.

**Figure 2:**
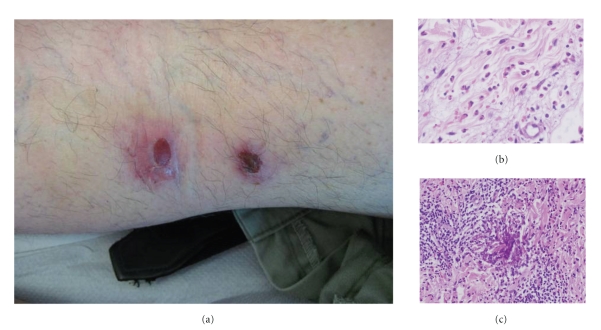
(a) Prurigo-like lesions with central crust mimicking arthropod bites. (b) Interstitial dermal infiltration composed by eosinophils. (c) Flame figures.

During successive outbreaks the lesions changed their morphology to urticarial, prurigo-like lesions resembling insect bites. Examination of skin revealed a widespread eruption consisting of erythematous papules, some with central crust and excoriations (Figure 2(a)). He denied history of arthropod assaults. A skin biopsy specimen showed a dense eosinophilic interstitial infiltrate that affected dermis (Figure 2(b)) with occasional “flame figures” (Figure 2(c)), and no changes in subcutaneous tissue were observed. The patient was diagnosed of insect bite-like reaction related to CLL. Skin lesions had a good response to topical and systemic steroid treatment, and recurrences were reduced with dapsone (150 mg daily). Cutaneous eruptions fully resolved when leukaemia was controlled. 

This case was classified as an eosinophilic dermatosis associated with lymphoproliferative disease that began as eosinophilic panniculitis and later evolved into lesions characteristic of insect bite-like reaction.

## 3. Discussion

Cutaneous findings are relatively common in patients with hematologic disorders [3–6]. In 1965, Weed described a peculiar cutaneous eruption in haematological patients and termed it “exaggerated delayed hypersensitivity to mosquito bites in CLL”. He demonstrated positive results in an intradermal test made with mosquito antigen and considered that these patients had an abnormal response to insect bites [7]. Since then, this disorder has been reported in the literature with different names, such as “insect bite-like reaction in hematologic neoplasm” [3, 8, 9]. However, most of these reports had not history, clinical picture, nor response to treatment or preventive measures suggestive of insect bite [5–8, 10], and terms like “eosinophilic dermatosis of myeloproliferative diseases” or “eosinophilic eruption of haematoproliferative diseases” were proposed [4, 9].

The exact pathogenesis of this disorder remains unclear. Skin eruption had features similar to some cutaneous reactions reported in patients with congenital agammaglobulinaemia and human immunodeficiency virus infection [6, 9, 11]. It is believed that in a patient with immunodeficiency due to hematologic disease a trigger like insect bite, drug, or virus induces cytokine production with an excess of interleukin 4 and 5 and an altered immune response with eosinophils predominance [4–6, 11, 12]. Depending on the localization of eosinophilic infiltrate in the skin the clinical manifestations ranged from papules to vesicles when infiltrate was superficial to panniculitis when subcutaneous tissue was affected. All of these different clinical presentations may be part of the same reactive spectrum. In our patient, the evolution of the lesions was peculiar. The unusual fact about our case was that the initial biopsy showed an eosinophilic panniculitis without an associated dermal element and the second biopsy revealed an eosinophilic infiltrate on dermis, and fatty tissue was normal.

This dermatosis should be included in the nonspecific eruption related to haematoproliferative disorders. It usually appears months to years after the diagnosis of neoplasm but sometimes may precede the diagnosis. In this manner, orientated investigations are recommended to detect a possible underlying haematological disease and to exclude a variety of other clinical conditions that may associate to eosinophilic infiltrate [4, 8, 9].

Treatment is difficult and recurrences are common. Systemic steroids are effective in suppressing eruptions, but lesions often recur when the dose is reduced. Chemotherapy regimens may also improve the lesions in some patients, but this is probably due to the fact that steroids are included in the regimen used [3, 8]. Dapsone and phototherapy can also prevent recurrences but cutaneous manifestations only disappear when the hematologic disease is controlled.

## 4. Conclusion

In summary, we report a patient with CLL who had outbreaks of cutaneous lesions with different clinical presentation (ranged from nodules to erythematous plaques) depending on the localization of eosinophilic infiltrate. We think these lesions may be manifestations of the same spectrum of eosinophilic dermatosis related to hematologic diseases.
